# In Vitro Enhanced Performance of Human Platelet Lysate Gel Integrated with Mesoporous Silica Nanoparticle/Carboxymethyl Chitosan Composite Hydrogel: Structural Stability and Biological Activities for Chronic Wound Healing

**DOI:** 10.1021/acsomega.5c13494

**Published:** 2026-03-09

**Authors:** Tareerat Lertwimol, Suwitchaya Jankam, Setthawut Kitpakornsanti, Weerachai Singhatanadgit, Wanida Janvikul

**Affiliations:** † National Metal and Materials Technology Center, National Science and Technology Development Agency, Pathum Thani 12120, Thailand; ‡ Faculty of Dentistry and Research Unit in Mineralized Tissue Reconstruction, 37698 Thammasat University (Rangsit Campus), Pathum Thani 12121, Thailand

## Abstract

Treatment of chronic wounds is challenging due to a variety of interconnected intrinsic (patient-related) and extrinsic (external/local) factors that disrupt the normal, orderly process of wound repair. Although human platelet lysate (hPL)-based treatment offers a cost-effective option for chronic wound diseases, the inherent limitations of hPL gel (hPLG), particularly its diminished structural integrity and limited retention of growth factors (GFs), hinder its utility for treating chronic wounds. Therefore, a novel cross-linked plasma-treated mesoporous silica nanoparticle/carboxymethyl chitosan composite (xPMC)-embedded hPLG hydrogel (xPMC/hPLG), designed to prolong fibrin integrity and enhance local retention of bioactive factors, was developed, characterized, and assessed for fibrin structural integrity and biological performance, including cellular chemotaxis and proliferation. Microcomputed tomography (micro-CT) and scanning electron microscopy (SEM) analyses demonstrated that xPMC possessed high porosity and interconnectivity, with evenly distributed fibrin within the pores of the composite hydrogel. Compared with hPLG alone, finer fibrin fibers with increased density were observed in xPMC/hPLG. The observed interpenetrating network structure of xPMC/hPLG was associated with the significantly reduced in vitro degradation of the composite hydrogel. Moreover, it exhibited controlled local release of the total protein and key growth factors, i.e., platelet-derived growth factor-BB (PDGF-BB) and transforming growth factor-β1 (TGF-β1). Importantly, xPMC/hPLG more effectively enhanced the recruitment and proliferation of periodontal fibroblasts than hPLG. This superior induction of fibroblast recruitment was at least partly mediated by PDGF-BB. In conclusion, the developed xPMC/hPLG composite hydrogel demonstrated prolonged structural integrity, extended release of bioactive GFs, and enhanced cell migration and proliferation. Future in vivo studies will further validate this composite hydrogel for enhancing the success of chronic wound treatment.

## Graphical abstract

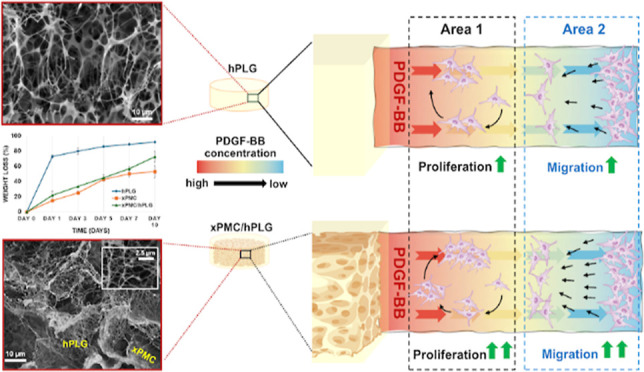

## 1 Introduction

The etiology of delayed or nonhealing chronic wounds is multifactorial, encompassing systemic conditions and local factors. Systemic diseases, such as diabetes and immunosuppression, represent significant impediments to physiological wound repair.^1–5^ The body’s immediate response to tissue damage initiates with hemostasis and inflammation, typically lasting for the first 3 days.^6,7^ During hemostasis, platelets activate, releasing growth factors (GFs) and various signaling molecules, while a fibrin network forms. This temporary scaffold provides structural support and acts as a storage depot for GFs released by platelets, which are essential for subsequent stages of healing.^8,9^ As inflammation begins, fibrinolysis, the enzymatic breakdown of the fibrin matrix, occurs. This process is crucial for dissolving the initial blood clot and maintaining open blood vessels, thereby ensuring proper blood flow.^10^ However, certain health issues, e.g., diabetes, liver disease, obesity, and blood clotting disorders,^2–5^ can lead to excessive fibrinolysis and changes in the structure and function of fibrin, hindering wound repair. For example, fibrin matrices with a thicker fiber structure are prone to rapid breakdown by fibrinolysis.^3–5,11^ Patients with nonhealing chronic wounds exhibit decreased cellular population and activity, as well as reduced production of structural extracellular matrix (ECM) components essential for wound healing.^1–5^ This suggests that manipulating the structure of fibrin in the early stages of wound healing could be a promising approach to control the rate and extent of blood clot degradation, ultimately promoting effective wound healing. In patients with difficult-to-heal wounds, whose repair potential is compromised, a reinforced, biocompatible GF-releasable hydrogel may help support prolonged matrix integrity and promote the migration and subsequent proliferation of resident cells to the wound site.

Stimulating resident cells to migrate to and proliferate at wound sites can be governed by chemokines^12,13^ that are abundant in platelet-derived products, including human platelet lysates (hPL).^14,15^ The use of hPL offers a cost-effective treatment option for chronic wound disease. This advantage stems from its provision of a concentrated “cocktail” of GFs that are immediately available upon application, thereby promoting accelerated healing and minimizing the total duration of treatment relative to conventional protocols.^16,17^

Transforming growth factor-β1 (TGF-β1) and platelet-derived growth factor-BB (PDGF-BB) are two key mediators among various hPL-derived GFs.^18,19^ In particular, the former represents the most abundant GF; the latter plays a dominant role in cell migration induced by platelets.^20–22^ Extensive research has explored the application of hPL gel (hPLG), both as a standalone matrix and in combination with other matrices, to support cellular expansion and proliferation.^23–25^ Subsequently, hPLG has been widely utilized as an encapsulation and delivery system for stem cells.^26,27^ However, the inherent limitations of hPL-based gels, particularly their diminished structural integrity and limited retention of GFs,^28^ suggest that they have restricted utility in treating difficult-to-heal chronic wounds.^29,30^

Among biomaterials classified by the United States Food and Drug Administration as “Generally Recognized As Safe (GRAS)” status for food and dietary supplements,^31,32^ carboxymethyl chitosan (CMC) and mesoporous silica nanoparticles (MSNs) are ones of good candidates to help improve structural integrity and local retention of GFs for the application of hPLG in treating chronic wounds. Beyond its inherent hydrophilicity, excellent biocompatibility, tunable biodegradability, antimicrobial properties, and hemostatic capability,^33,34^ the strong binding affinity of CMC for fibrinogen may facilitate the formation and stability of hPLG.^35^ The fibrinogen adsorption on MSNs may also hinder the formation of fibrin fiber chains through their porous structure,^36^ resulting in the formation of thin fibers and a dense gel structure that resists hPLG degradation.^37^ A preliminary study indicated that steam-cross-linked CMC was completely degraded within 4 days, which was insufficient time to support tissue repair.^38^ Particularly in chronic wounds, after implantation, the biomaterial must maintain appropriate structural integrity to support the attachment and growth of migratory resident cells.^39^ Chemical cross-linking is a better approach for slowing the degradation of cross-linked CMC.^40^ The conjugation of *N*-(3-(dimethylamino)­propyl)-*N*′-ethylcarbodiimide hydrochloride (EDC) and *N*-hydroxysuccinimide (NHS) (EDC/NHS) is often employed as a chemical cross-linking agent to enhance the physical stability of materials.^41^ Both CMC and MSNs can effectively absorb and adsorb, respectively, proteins,^42–44^ suitably enabling their use for the controlled release of hPL-derived GFs. The prevention of rapid GF diffusion from hPL, thereby, helps concentrate these beneficial molecules at the wound site.

In this study, we aimed to develop an hPL-based composite biomaterial to prolong fibrin integrity and enhance the local retention of bioactive factors. To achieve this, a porous composite sponge comprising carboxymethyl chitosan (CMC) and plasma-treated Mobil Composition of Matter No. 41 (MCM-41) mesoporous silica nanoparticles (coded as PM) was initially fabricated and then cross-linked using EDC/NHS chemistry. The resulting cross-linked CMC/PM composite material was subsequently incorporated into hPL prior to gelation using calcium chloride solution. Both composite-free and composite-embedded hPLG hydrogels were compared in terms of fibrin structural integrity and biological performance, including cellular chemotaxis and proliferation.

## 2 Materials and Methods

### 2.1 Materials

Water-soluble carboxymethyl chitosan (CMC) (*M̅*_w_ = 3.0 × 10^5^ Da, degree of substitution (DS) = 0.9) and plasma-treated MCM-41 mesoporous silica nanomaterial (PM) were prepared in our laboratory, following the procedure described in our previous work.^45^ Absolute ethanol (EtOH) was obtained from CT Chemical Co., Ltd. (Thailand). *N*-(3-(dimethylamino)­propyl)-N′-ethylcarbodiimide hydrochloride (EDC, Cat. No. 03450) and *N*-hydroxysuccinimide (NHS, Cat. No. 56480) were purchased from Sigma and Fluka, respectively. All analytical-grade chemicals were used as received without further purification.

### 2.2 Preparation of Human Platelet Lysate (hPL) and Human Platelet Lysate Gel (hPLG)

The hPL sample was prepared from outdated pooled leukocyte-poor platelet concentrates (LPPC), which were kindly provided by the Thammasat University Hospital Blood Bank with approval from the Ethics Review Sub-Committee for Research Involving Human Research Subjects of Thammasat University No. 3 (120/2566) and the Institutional Biosafety Committee of Thammasat University (012/2567). Each LPPC containing approximately 1 × 10^9^ platelets/mL was subjected to three freeze/thaw cycles (freezing at −80 °C and thawing at 37 °C), followed by centrifugation at 4000*g* for 15 min at 4 °C to remove debris. The obtained supernatant was subsequently collected, passed through a 0.22 μm syringe filter, and stored at −80 °C until use.

To form hPLG, 1 M CaCl_2_ solution was added to 1 mL of hPL to reach a final concentration of 25 mM. A 45 μL mixture was pipetted into an open-ended cylindrical polypropylene (PP) mold (6 mm diameter x 5 mm height), and the sample was then allowed to gel at 37 °C for 1 h. The entire preparation process is schematically depicted in Scheme 1A.

**Scheme 1:**
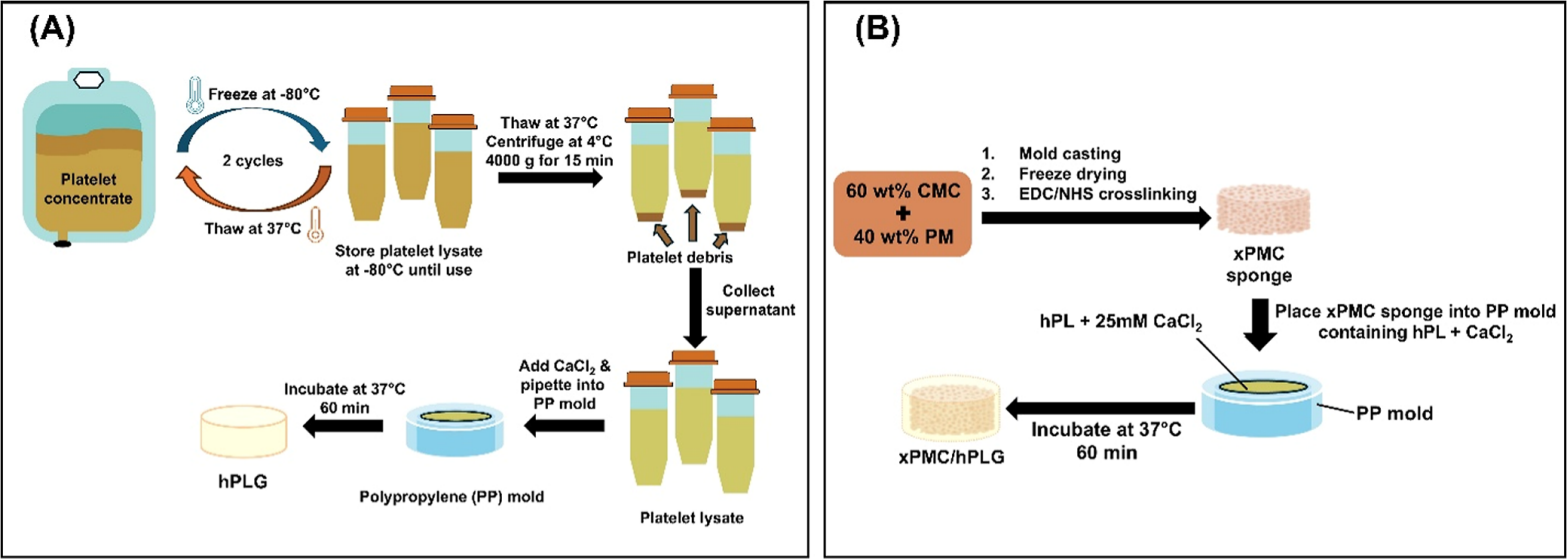
Schematic Illustration of Preparations of (A) hPLG and (B) xPMC/hPLG

### 2.3 Preparation of Cross-Linked Composite-Embedded hPL Hydrogel (xPMC/hPLG)

MCM-41 nanoparticles were subjected to a 1 h low-pressure oxygen plasma treatment using a 13.56 MHz inductively coupled radio frequency plasma, following established protocols,^46^ yielding plasma-treated MCM-41 (PM). The physicochemical attributes of PM, encompassing crystalline phase structure (elucidated via X-ray diffraction), specific surface area and porosity (quantified by Brunauer–Emmett–Teller analysis), and elemental composition and surface chemical states (determined through X-ray photoelectron spectroscopy), were thoroughly characterized and previously reported. Following this, 300 mg of carboxymethyl chitosan (CMC) was mixed with 200 mg of PM in 10 mL of deionized water at ambient temperature and agitated for 24 h. The resulting mixture was then cast into a rectangular mold with dimensions of 7 (length) × 5 (width) × 0.2 (height) cm^3^. The cast material was subsequently freeze-dried, resulting in a 40/60 w/w PM/CMC composite pad (designated as PMC) with a total solid content of 5 wt %. The dried PMC pad was cut and weighed to approximately 100 mg prior to chemical cross-linking (details provided in Table 1). Later, the cross-linked hydrogel pad (denoted as xPMC) was successively washed with deionized water to remove residual cross-linking reagents, freeze-dried, and cut into small disks with a diameter of 4 mm.

**Table 1:** Crosslinking of the PMC Pad Using EDC/NHS Coupling Agents

	cross-linking condition				
PMC pad (mg)	EDC (mM)	NHS (mM)	DI (mL)	time (h)	temperature (°C)
100	15	12.7	30	16	room temperature

Typically, a UV-sterilized cylindrical xPMC disk with an average dry weight of 1.5 ± 0.3 mg was placed into a polypropylene mold containing 45 μL of the mixture of hPL and 25 mM CaCl_2_, at a 1:1 dry mass ratio of xPMC to hPL. The xPMC specimen was initially gently squeezed to eliminate air bubbles and allow xPMC to absorb hPL into its internal pores. The mixture of xPMC and hPL (coded as xPMC/hPL) was subsequently incubated for 1 h at 37 °C for gel formation, generating the composite-embedded hPL gel (xPMC/hPLG). The complete preparation process is schematically illustrated in Scheme 1B.

### 2.4 Characterization of Morphologies and Structures of xPMC and xPMC/hPLG Hydrogels

The microstructure of xPMC pores was analyzed using X-ray microcomputed tomography (μCT). First, the xPMC hydrogel was fully hydrated in deionized water, followed by Lugol’s iodine staining for 24 h. The stained sample was scanned using a μCT SkyScan 1275 (Bruker μCT, Kontich, Belgium) under the following parameters: pixel size = 8 μm, source voltage = 52 kV, source current = 95 μA, no filter, and rotation step = 0.2°. Visualizations were acquired using a CTVox (3D images, Bruker). The data sets were binarized using an adaptive threshold to distinguish dense material regions from voids, and despeckling operations in 3D were applied to reduce image noise. Porosity analysis was performed using the CTAn software (Bruker), and interconnectivity was calculated as the percentage of open pore space volume to the total pore space volume. The total porosity (%) and interconnectivity (%) are obtained as mean ± SD based on the analysis of the middle and peripheral regions (thickness = 1 mm) of a hydrogel specimen.

To observe the distribution of hPL-derived fibrin within xPMC pores, the cross-sectional morphology of the xPMC/hPLG hydrogel was examined by a scanning electron microscope (Neoscope JCM-6000 plus Joel benchtop SEM, Japan) in comparison with that of the composite-free hPLG hydrogel. Before SEM analysis, the freeze-dried specimens were attached to a conductive carbon tape and then coated with gold using a sputter coater at 15 mA for 180 s.

The SEM images were analyzed using open-source software ImageJ. The analysis included quantification of randomly selected xPMC pore sizes (*n* = 125 pores) and fiber diameters (*n* = 250 fibers). In addition, fibrin network density was determined by calculating the percentage of the region of interest (ROI) occupied by fibers.^47^ At least 10 ROIs were analyzed per sample using the following formula: % Fibrin density = (Fiber Area/ROI Area) × 100, where ROIs were created in ImageJ by selecting the Rectangular ROI tool from the toolbar and dragging it over the area of interest in the image.

### 2.5 In Vitro Degradation Test

To evaluate the biodegradability of materials, hPLG and xPMC/hPLG specimens with a similar cylindrical shape were separately immersed in phosphate-buffered saline (PBS) (pH 7.4) containing 0.2 mg/mL lysozyme to mimic accelerated hydrolysis conditions.^48^ At predetermined time points, the samples were removed, freeze-dried, and weighed. The percentage of weight loss is calculated as follows: Weight loss (%) = 100­[(*W*_0_ – *W*_t_)/*W*_0_], where *W*_0_ is the initial weight of the specimen, and *W*_t_ is the weight of the specimen after being immersed for a given period.

To confirm the degradation determined by a gravimetric method, the autofluorescence of xPMC/hPLG was measured in parallel at Days 0, 1, 5, and 10. For Day 0, after gel formation, the xPMC/hPLG specimen was soaked in lysozyme solution for 3 h at 37 °C before imaging. All specimens were visualized and captured using a Leica DM IL LED inverted microscope (Leica Microsystems, Switzerland) via fluorescence mode. The center of the top surface of each specimen was observed in two channels (green (525 nm) and blue (462 nm)) using the same exposure time and gain settings, and the merged dual-color autofluorescence image was then recorded.

### 2.6 Analysis of Total Protein, TGF-β1 and PDGF-BB

To determine the release of total protein, TGF-β1 and PDGF-BB, hPLG and xPMC/hPLG samples, composed of an equal total amount of hPL, were individually placed in a 48-well plate containing 0.35 mL of PBS and incubated at 37 °C; the PBS solution was collected daily and replaced. The withdrawn PBS samples were kept at −20 °C until analysis. To examine the protein composition, the samples were mixed separately with loading dye (Bio-Rad, CA, USA) and then heated at 95 °C for 5 min to denature. A protein ladder (Bio-Rad, CA, USA) and 20 μL of each sample were loaded into separate lanes of a 10% polyacrylamide gel. After electrophoresis, the gel was stained with Coomassie Blue G-250 (Bio-Rad, CA, USA) to visualize proteins of different sizes in the samples. For quantification of total protein, TGF-β1 and PDGF-BB, the Pierce BCA Protein Assay Kit (Thermo Scientific, USA) and ELISA kits for TGF-β1 and PDGF-BB (R & D Systems, Canada) were used by following the manufacturer’s instructions.

The ability of PM and cross-linked CMC (xCMC) to adsorb and absorb, respectively, proteins released from hPLG was also investigated. xCMC was prepared from 100% CMC and cross-linked using EDC/NHS, following the same procedure as for xPMC. 1.5 mg of PM or xCMC was added to 0.5 mL of PBS containing hPLG. A control sample without either material was also prepared. All samples were incubated at 37 °C for 48 h. Following incubation, the remaining protein content in each PBS solution was quantified using the BCA assay, as described above.

### 2.7 Isolation and Culture of Human Periodontal Fibroblasts

In the present study, human periodontal fibroblasts, isolated from human periodontal ligament (PDL) tissue, were used following approval by the Ethics Review Sub-Committee for Research Involving Human Research Subjects of Thammasat University No. 3 (120/2566) and the Institutional Biosafety Committee of Thammasat University (012/2567). The PDL tissue was isolated from the middle region of the root surface of extracted teeth obtained from healthy donors. The cells were explanted from the PDL tissue and cultured in high-glucose Dulbecco’s modified Eagle’s medium (DMEM) supplemented with 10% heat-inactivated fetal bovine serum (FBS) (Gibco), 1% antibiotic-antimycotic reagent (Gibco), and 1% Glutamax (Gibco) (standard culture medium). The medium was refreshed every 2–3 days, and cells were passaged using 0.25% trypsin–EDTA solution (Gibco) after reaching 80% confluency. Cells between passages 5–8 were used in the present study.

### 2.8 Fibrinogen-Depleted Human Platelet Lysate (FD-hPL) Treatment of Cells

FD-hPL was prepared by removing fibrinogen from hPL through calcium-induced clotting, as reported previously.^49,50^ Different FD-hPL-supplemented media containing FD-hPL at 10%, 25%, 50%, and 75% and FBS at 2% in the standard culture medium were freshly prepared and used to treat the cells. Briefly, PDL cells were first seeded on a 96-well plate at a density of 2 × 10^3^ cells/well in 0.2 mL of standard culture medium containing 2% heat-inactivated FBS at 37 °C for 24 h. Subsequently, the cells were treated with each FD-hPL-supplemented medium. After a 24 h treatment period, each sample was subjected to the Alamar Blue assay and live/dead cell staining, as described below.

### 2.9 Cell Seeding and Culture on hPLG and xPMC/hPLG Hydrogels

To evaluate the effects of hPLG and xPMC/hPLG hydrogels on the metabolic activity and growth of periodontal fibroblasts, the hydrogels were prepared according to Sections 2.3. Cells were then seeded onto these samples in 48-well plates at a density of 1 × 10^4^ cells/well in 0.3 mL of standard culture medium containing 2% heat-inactivated FBS. After incubation periods of 1, 2, and 3 days, the cell-seeded specimens were transferred to new wells and subjected to the Alamar Blue assay for the determination of metabolic activity and live/dead cell staining for the determination of viability and population doubling time.

### 2.10 Alamar Blue Assay

The metabolic activity of cells grown on hPLG and xPMC/hPLG was determined using the Alamar Blue assay. Following treatment of cells as described above, fresh standard culture medium containing 0.5 mM Alamar Blue solution was added to each well and incubated for 4 h. The fluorescence intensity was measured in a multilabel plate reader (VICTOR X4, PerkinElmer, USA) using an excitation wavelength of 530 nm and an emission wavelength of 590 nm. The fluorescence intensity is directly proportional to cellular metabolic activity, with 100% activity defined by the untreated control group.

### 2.11 Live/Dead Cell Staining Assay

The viability and population doubling time of cells grown on hPLG and xPMC/hPLG were determined using the LIVE/DEAD Viability/Cytotoxicity Kit (Invitrogen, USA) according to the manufacturer’s protocol. Briefly, the cells were washed with PBS and incubated with serum-free DMEM containing 4 μM calcein-AM and 2 μM ethidium homodimer at 37 °C for 20 min. After a further PBS wash, labeled cells were visualized and captured under an inverted fluorescence microscope (Leica Microsystems, Switzerland). Live cells are stained green, and dead cells are stained red. Population doubling times (PDT) were calculated from direct cell counts obtained from the live/dead staining images at each experimental time point. The PDT was obtained using the formula: PDT = log_2_(Δ*t*)/(log­(*N*) – log­(*N*_0_)), where Δ*t* represents the growth time (h); *N* is the total number of live cells at each time point, and *N*_0_ is the initial number of cells seeded onto each material. Additionally, the number of dead cells was quantified and compared across groups at each measurement time point.

### 2.12 Cell Migration Assay

Cell migration in response to the tested chemokine was evaluated using the agarose spot assay.^51^ In this study, we performed gel spot adapted from agarose spot by replacing the droplet of agarose containing different chemoattractants with the spots of hPLG and xPMC/hPLG for migration analysis in comparison with those of human platelet-poor plasma gel (hPPG), plasma with a very low number of platelets, used as a control containing less chemoattractant. A 10 μL of platelet-poor plasma (PPP) or hPL (containing 25 mM CaCl_2_) was spotted onto the center of each well of a 48-well plate. The droplet of xPMC/hPLG was prepared by cutting the sterile xPMC into small spherical pieces with an approximate dimension of 1 mm (diameter) × 2 mm (height). A single droplet of xPMC/hPLG contained 2 pieces of xPMC mixed with 10 μL of hPL/CaCl_2_ before dropping, maintaining approximately a 1:1 dry mass ratio of xPMC to hPL. All gel spots were allowed to polymerize at 37 °C for 60 min. Cells were seeded on each well with a gel spot at a density of 1 × 10^4^ cells/well/0.3 mL. The cells were cultured in DMEM + 1% antibiotic–antibiotic agent without serum supplementation for 6 h. At the end of the incubation period, the medium was removed, and the cells were then fixed with 4% paraformaldehyde/PBS at 4 °C overnight. The fixed cells were stained with 0.2% w/v crystal violet for 30 min and gently washed with PBS four times (10 min each) to eliminate the excess dye. Cell migration can be observed microscopically and captured using an inverted microscope (Leica Microsystems, Switzerland). To quantify and compare migration between groups, the number of cells that had migrated near the edges of the sphere within a 500 μm radial distance was counted and reported as cells/mm^2^. Four positions of each gel were captured (5× objective).

To unequivocally determine the role of PDGF-BB specifically as a primary GF mediating hPL-induced periodontal fibroblast migration, PDGF-BB activity was inhibited by preincubating xPMC/hPLG spots with 0.2 μg/mL of a PDGF-BB neutralizing antibody (AF-220-NA, R&D Systems) for 45 min. As a negative control, xPMC/hPLG spots were pretreated for 45 min with an isotype control goat IgG antibody (0.2 μg/mL; AB-108 C, R&D Systems).

### 2.13 Statistical Analyses

Statistical analyses were performed using SPSS software (version 19.0; SPSS, Inc., Chicago, IL). For pairwise comparisons between two independent groups at each time point, an independent-samples *t*-test was conducted, using a one-tailed hypothesis. Statistical significance was defined as *p < 0.05 and **p < 0.01. For multiple comparisons following a significant one-way analysis of variance (ANOVA), Duncan’s multiple range test was applied. Significant differences between groups are indicated by distinct letters, where *p* < 0.05.

## 3 Results and Discussion

### 3.1 Characterization of Cross-Linked Composite-Free and Composite-Embedded hPL Hydrogels

The porous xPMC composite pad was prepared by combining plasma-treated MCM-41 nanoparticles (PM) and carboxymethyl chitosan (CMC) at a 40:60 weight ratio, followed by cross-linking through an EDC/NHS reaction. Figure 1A,B present top surface and cross-sectional microcomputed tomography (μCT) images of the fully hydrated xPMC hydrogel, respectively. The resulting 3D architecture exhibited a highly interconnected porous structure with a porosity of 69.2 ± 8.9% and an interconnectivity of 99.9 ± 0.1%. The average pore size of the freeze-dried xPMC, measured directly from its SEM images (Figure 1C) using ImageJ software, was approximately 206 ± 72 μm (*n* = 5 images, 25 pores per image). The fibrin architecture of hPLG gelled with calcium chloride displayed an overlapping and entangled mesh structure (Figure 1D). The SEM image of xPMC/hPLG in Figure 1E shows that fibrin derived from hPL was evenly distributed within the pores of the xPMC hydrogel. In addition, the fibrin network formed within the xPMC structure appeared finer than that observed in pristine hPLG (Figure 1F,G). The diameter of fibrin fibers within the xPMC pores (176 ± 49 nm) was significantly thinner than that of fibrin fibers in hPLG (523 ± 316 nm), determined by measuring fiber diameters (*n* = 250) from SEM images using ImageJ (*p* < 0.01) (Figure 1H). Fibrin density (%) was quantified from the SEM images by determining the percentage of the region of interest (ROI) area occupied by fibrin fibers using ImageJ software. The fibrin fibers formed within the xPMC/hPLG pores were significantly denser than those found in hPLG, with average fiber areas of 74% and 58%, respectively (Figure 1I).

**Figure 1:**
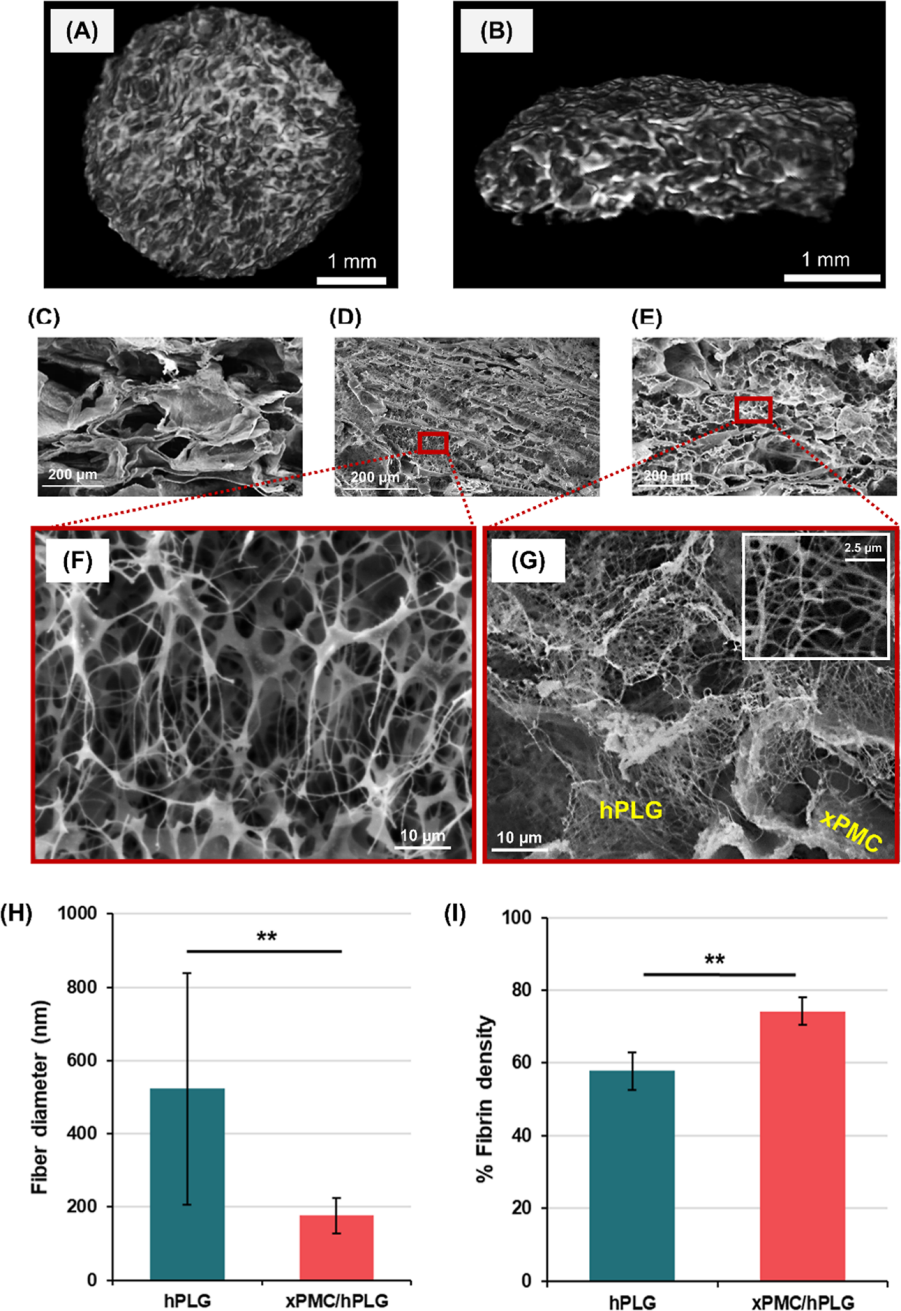
Porous structures and morphologies of the prepared samples. Micro-CT images showing the 3D porous architecture of the fully hydrated xPMC hydrogel in (A) the top and (B) cross-sectional views. SEM images of cross-sectional morphologies of (C) xPMC, (D) hPLG, (E) xPMC/hPLG, and magnified SEM images of (F) hPLG and (G) xPMC/hPLG structures. The inset in (G) displays fine fibrin fibers at high magnification. (H) Fibrin fiber diameter measured from SEM images via ImageJ (**p < 0.01) (*n* = 125). (I) Fibrin density (%) determined from SEM images by measuring area coverage by fibrin (**p < 0.01), using at least 10 regions of interest (ROIs) per sample. Data are represented as mean ± SD.

The highly interconnected porous structure of the prepared xPMC composite hydrogel, consisting of PM and xCMC, may be suitable for tissue ingrowth and regeneration.^52,53^ There existed a homogeneously integrated PL-derived fibrin network in the xPMC pore structure of the xPMC/hPLG hydrogel with a reduced fibrin fiber diameter and a concomitantly increased fibrin density compared with the randomly oriented fibrin network observed in the original hPLG. The observed physical distinctions suggested that xPMC affected both the mechanisms of fibrinogen assembly and the structure of the resulting fibrin network. Specifically, the positively charged CMC surface bound with negatively charged fibrinogen primarily via electrostatic interactions,^35^ thereby concentrating fibrinogen within the confined pore space and subsequently leading to the generation of dense fibrin fibers. Furthermore, this phenomenon might also be attributed to the adsorption of fibrinogen onto the PM nanoparticles present in the xPMC hydrogel. This adsorption likely hindered the formation of fiber chains within the pores of PM,^36^ resulting in thinner and denser fibers, compared with those observed in hPLG. The precise mechanism by which xPMC regulates PL-derived fibrin architecture and its clinical significance warrant further study.

### 3.2 Degradability of hPLG, xPMC and xPMC/hPLG Hydrogels

In vitro degradation of the prepared hydrogels was comparatively monitored over a 10 day incubation period at 37 °C in phosphate-buffered saline (PBS) containing 0.2 mg/mL lysozyme. Figure 2A shows the weight loss profiles of hPLG, xPMC and xPMC/hPLG hydrogels. The hPLG hydrogel exhibited the highest degradability, with a weight loss of 73% after 24 h incubation in a lysozyme solution; thereafter, its mass loss rate gradually slowed until Day 10, with a final weight loss of 92%. The observed rapid degradation profile of hPLG was in accordance with findings previously reported.^29,54^ Conversely, the xPMC hydrogel exhibited the slowest degradability; an initial weight loss of 15% was observed on Day 1. The degradation of this composite hydrogel, primarily derived from the organic component, i.e., cross-linked CMC (xCMC), appeared rather steady with approximately 53% remaining mass found after being soaked in the lysozyme solution for 10 days, implying that about 80% of xCMC in the composite had been degraded. Given that the xPMC specimen was produced via chemical cross-linking (EDC/NHS reaction), the weight loss at the early stage (Day 1) was likely due to the release of the non-cross-linked CMC component, followed by steady degradation mediated by lysozyme activity. Unexpectedly, the degradation profile of the xPMC/hPLG hydrogel, composed of a 1:1 dry weight ratio of xPMC and hPLG, did not exhibit an intermediate behavior between those of pristine hPLG and xPMC hydrogels; it was rather similar to that of xPMC, but with a slightly greater cumulative weight loss on Day 10. Evidently, the incorporation of xPMC into hPLG not only distinctly decelerated the degradation but also improved the stability of the hydrogel; therefore, the sustained release of GFs from the composite-embedded hPLG hydrogel was anticipated, thereby establishing an appropriate localized delivery system.

**Figure 2:**
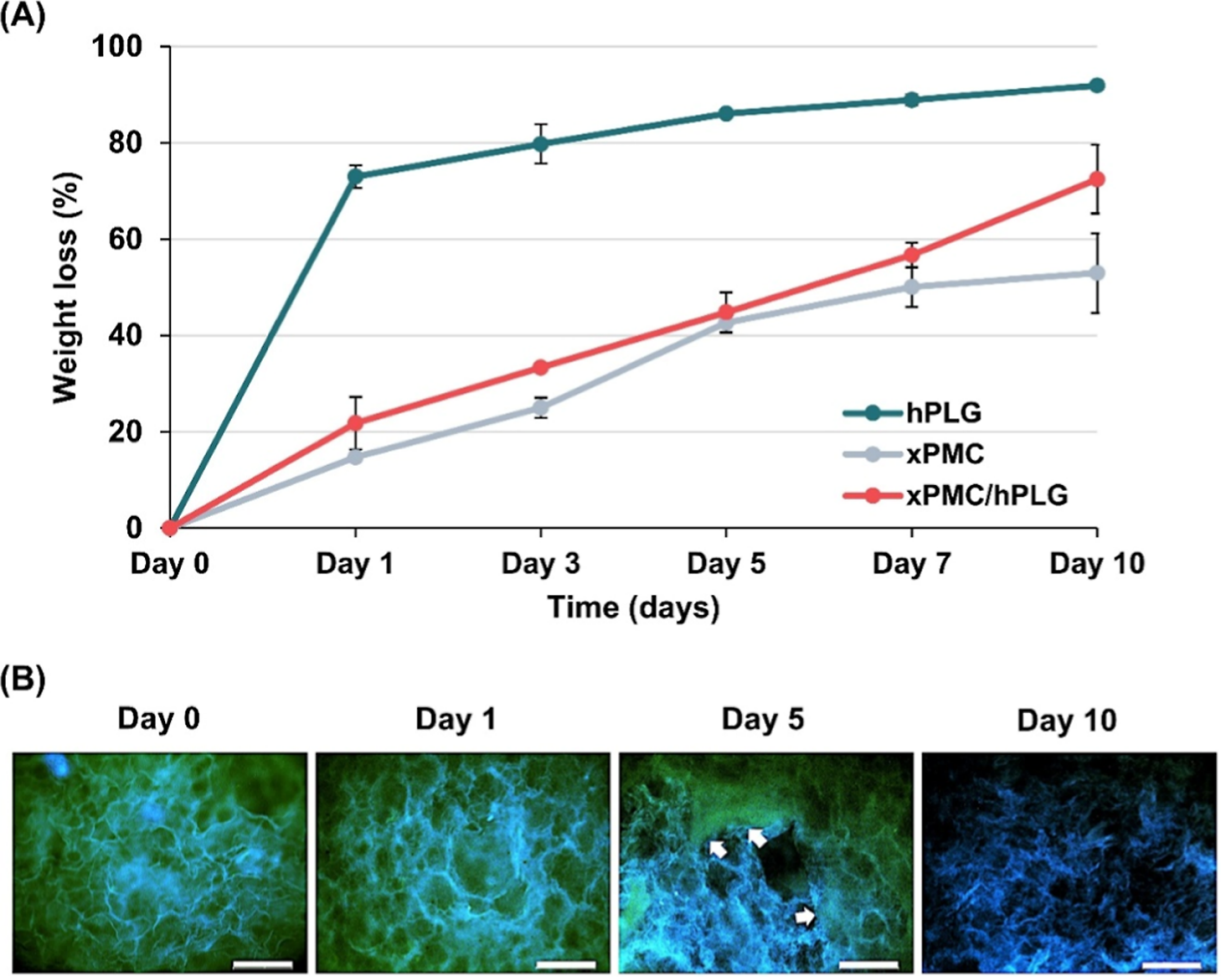
Degradation of hPLG, xPMC, and xPMC/hPLG after being individually immersed in PBS containing 0.2 mg/mL lysozyme at 37 °C for 1, 3, 5, 7, and 10 days. (A) Weight loss changes were determined. The results are expressed as mean percentage ±SD (*n* = 3). (B) A qualitative analysis of fibrin density changes during degradation, observed using inverted fluorescence microscopy, was performed. Autofluorescence of the top surface of the xPMC/hPLG hydrogel, observed in the green (fibrin) and blue (xPMC) emission channels, was captured using the same microscopy settings. White arrows indicate the extent of hPLG coverage on the xPMC- embedded hydrogel. Scale bar = 500 μm.

To follow the degradation profile of the xPMC/hPLG hydrogel, the autofluorescence of fibrin (green) and xPMC (blue) was observed from the top surface of each test specimen after being incubated in the lysozyme solution for a given period. Unstained hPLG-derived fibrin exhibited an autofluorescence signal in the green channel (Figure S1), which was consistent with the previously reported results.^38,55^ Furthermore, it has been reported that CMC exhibited a higher fluorescence intensity than chitosan on the blue channel. This increased fluorescence intensity of CMC is positively correlated with its greater mass, due to the presence of carboxymethyl groups.^56^ Figure 2B vividly reveals the green fibrin signal densely surrounding the inserted xPMC hydrogel (observed under blue light) on Day 0. The nearly negligible changes in fibrin structure were observed during the initial 24 h of immersion in the lysozyme solution; meanwhile, the autofluorescence of xPMC still exhibited an intact honeycomb-like pore structure with a larger pore size than that observed on Day 0, indicating xPMC swelling during this immersion period. After 5 days of incubation, the xPMC/hPLG surface revealed the marked degradation of the covered hPLG hydrogel (indicated by white arrows), suggesting that the weight loss of xPMC/hPLG was predominantly derived from hPLG rather than xCMC in xPMC; despite the almost equal percentages of weight loss of both xPMC/hPLG and xPMC, as shown in Figure 2A. The surrounding hPLG material was completely degraded by the enzymatic action of lysozyme within 10 days, resulting in compromised structural integrity of the xPMC pores. Consequently, xPMC/hPLG exhibited greater weight loss on Day 10 than xPMC (Figure 2A).

The prepared xPMC/hPLG hydrogel could deaccelerate the rapid degradation that typically occurs within a few days for hPLG.^30^ An entangled interpenetrating polymer network (IPN) formed between the cross-linked xPMC network and the hPLG-derived fibrin network played a crucial role in enhancing degradation resistance. Clinically, the xPMC/hPLG hydrogel may enhance the stability of nascent healing tissue at the wound site, particularly in severely inflamed areas characterized by a significant accumulation of inflammatory cells that produce proteolytic enzymes.

Microbial infection also significantly impedes the healing of chronic wounds. Bacteria not only release cytotoxic substances that damage wound site cellular components, but they also secrete proteases that accelerate the degradation of fibrinogen/fibrin^57^ and collagen.^58^ This enzymatic activity severely compromises the structural integrity of the wound matrix. Effective chronic wound management, therefore, necessitates strategies that preserve matrix integrity, thereby fostering cellular proliferation and function. A potential therapeutic mechanism of the xPMC/hPLG hydrogel may help maintain matrix integrity through the interaction of the hydrogel components with the fibrin mentioned above. The enzymatic hydrolysis of CMC yields chitooligosaccharides (COS). COS are known to inhibit the activity of lysozyme^59^ and matrix metalloproteinases (MMPs),^60^ key enzymes responsible for degrading the extracellular matrix (ECM) during wound healing. This inhibitory effect of COS may also contribute to enhanced fibrin network stabilization, as observed in the xPMC/hPLG hydrogel (Figure 2). Beyond enzyme inhibition and fibrin stabilization, the incorporation of the xPMC hydrogel might offer additional therapeutic advantages for chronic wound treatment. COS exhibits antioxidant, anti-inflammatory, and antimicrobial activities,^60,61^ all of which are beneficial in the complex environment of a chronic wound. However, further study is needed to confirm the presence of COS derived from the hydrolysis of CMC in xPMC/hPLG and its possible contribution in fibrin integrity.

### 3.3 Controlled Release of Total Protein and GFs

Several hPL-derived GFs play a crucial role in cell signaling by acting as chemoattractants, molecules that attract cells and direct their movement. To enhance cell-recruiting capacity and promote cell growth, both hPLG and xPMC/hPLG hydrogels prepared in this study were used for the delivery of GFs. Therefore, analyzing the protein release profiles from individual hydrogel specimens is essential for advancing our understanding of their function. The release of total protein from each hydrogel was daily evaluated using the BCA assay under physiological-like conditions for up to 4 days. The cumulative release profiles of total protein from hPLG and xPMC/hPLG hydrogels are comparatively shown in Figure 3A. At 24 h postincubation, the total protein liberated from hPLG and xPMC/hPLG was 74% and 67%, respectively, and increased to 91% and 78%, respectively, over the subsequent 4 day period. Additionally, the collected supernatants released from both samples were analyzed by SDS-PAGE, followed by Coomassie blue staining, as shown in Figure 3B. The most abundant zone appeared at ∼66 kDa, corresponding to the albumin band derived from the major plasma protein, which was most prominent in the supernatant obtained from hPL.^62^ Overall, the protein bands observed in the xPMC/hPLG supernatant exhibited reduced intensity over time compared with those in the hPLG releasate. These findings suggested that the xPMC/hPLG hydrogel facilitated a relatively slower release of the total protein.

**Figure 3:**
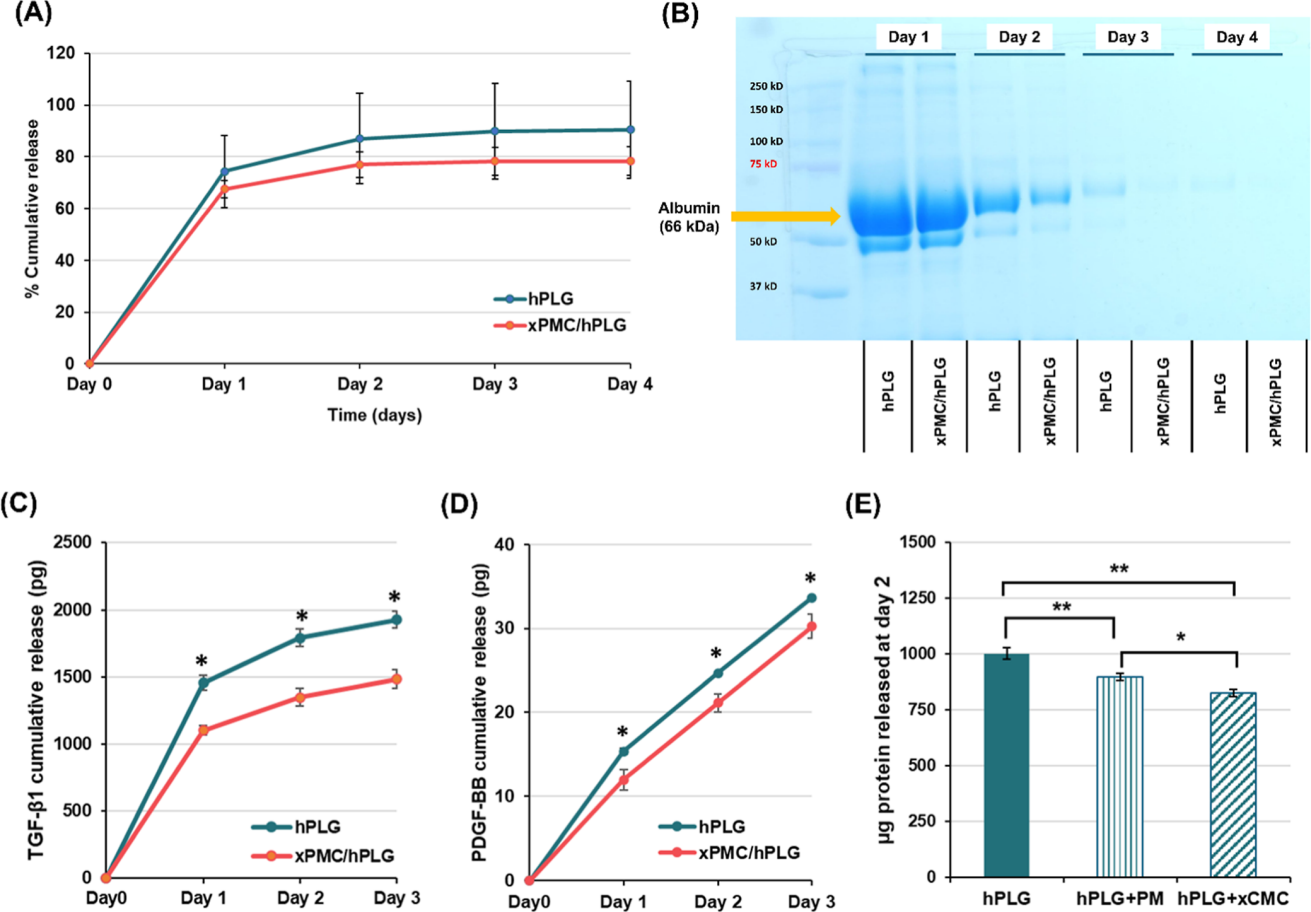
Measurements of total protein and growth factors released from hPLG and xPMC/hPLG. (A) Cumulative release percentage of total protein over a 4 day period, determined by BCA assay. (B) SDS-PAGE analysis results of the PBS supernatants collected over a 4 day period, stained with Coomassie blue. Cumulative release of (C) TGF-β1 and (D) PDGF-BB over a 3 day period. (E) Total protein contents found in the PBS supernatants of hPLG and hPLG combined with either PM or xCMC, assessed after a 2 day incubation. Data are expressed as mean ± SD (*n* = 3). *p < 0.05, **p < 0.01. Statistical significance in (C,D) was analyzed at the same time point.

The cumulative releases of the hPL-derived GFs involved in cell migration and viability, that is, TGF-β1 and PDGF-BB,^22,63,64^ were quantified, and analysis of cumulative TGF-β1 release demonstrated an initial burst release in both hPLG and xPMC/hPLG groups on Day 1 (Figure 3C). However, the xPMC/hPLG hydrogel exhibited a statistically significant reduction (*p* < 0.05) in the cumulative TGF-β1 release over 3 days, compared with the hPLG group. These findings indicated that the xPMC/hPLG hydrogel facilitated a more controlled TGF-β1 release profile than hPLG alone. Figure 3D illustrates the daily cumulative release of PDGF-BB from hPLG and xPMC/hPLG hydrogels for 3 days. On Day 1, the hPLG group revealed a statistically significant higher release of PDGF-BB, compared to the xPMC/hPLG group (*p* < 0.05).

To evaluate the protein adsorption capacity of xCMC and PM, hPLG was prepared and immersed in PBS buffer under three conditions: with 1.5 mg PM (coded as hPLG + PM), with 1.5 mg xCMC (coded as hPLG + xCMC), and without either (used as a control). Following a 48 h incubation period, the remaining protein content released from hPLG impregnated in each condition was determined using a BCA assay. Both hPLG + PM and hPLG + xCMC showed significant reductions in protein content levels (*p* < 0.01), compared with the hPLG control, confirming the protein adsorption capabilities of both xCMC and PM (Figure 3E). The protein reduction was also statistically significant (*p* < 0.05) when comparing hPLG + xCMC to hPLG + PM. This suggested that xCMC played a superior role in protein adsorption than PM. Overall, the xPMC-embedded hPLG hydrogel enabled the controlled release of both TGF-β1 and PDGF-BB, compared with hPLG alone.

The xPMC specimen in xPMC/hPLG further facilitated the controlled release of bioactive molecules through a diffusion-based mechanism within its polymeric matrix. This controlled delivery enabled these molecules to precisely target cells within the surrounding microenvironment, thereby contributing to the overall therapeutic efficacy. The results showed that compared with hPLG, xPMC/hPLG allowed a slower release of the total protein and two established platelet-associated GFs, i.e., TGF-β1 and PDGF-BB (Figure 3). The former represents the most abundant GF, and the latter plays a dominant role in cell migration induced by platelets.^20–22^ The release profiles of these two GFs from both hPLG and xPMC/hPLG were characterized by a high initial release within the first 24 h, followed by a gradual decrease in release. This phenomenon may be attributed to the heterogeneous distribution of GFs within the matrix, as previously reported by Jalowiec et al.^65^ GFs located at the periphery of the matrix are likely to exhibit faster diffusion rates, compared with those located centrally. Notably, the slower and lower releases of TGF-β1 and PDGF-BB are added value of the incorporation of xPMC into hPLG. This may be caused by the potential retention of these hPL-derived GFs within the xPMC/hPLG hydrogel through its previously reported binding affinity for fibrinogen/fibrin, as well as its physical entrapment within the hydrogel matrix.^66^ Additionally, prior research suggested that a hydrogel matrix with a higher fibril density and finer fibril structure, as observed in xPMC/hPLG (Figure 1F–I), provided enhanced structural support for GF immobilization, thereby improving its retention.^37,67^ It is also possible that given the xCMC and PM composition, the interactions between these components and GFs could occur through a variety of mechanisms, including electrostatic interactions or hydrogen bonding with CMC^35,68,69^ and surface-modified (e.g., plasma-treated) PM.^36,70^ These possibilities were at least partly supported by the present findings shown in Figure 3E. Beyond the adsorption of individual components, the interpenetrating polymer network (IPN) of xPMC/hPLG might also contribute to GF retention within the material, as shown previously for the semi-IPNs of fibrin from autologous leukocyte- and platelet-rich plasma (L-PRP) and hyaluronic acid (HA), which exhibited significantly lower GF release than fibrin alone. The dense network packing of the semi-IPNs acted as a diffusive barrier.^71^

### 3.4 Metabolic Activity, Doubling Time, and Viability of Periodontal Fibroblasts

Since several key GFs in hPL, including TGF-β1 and PDGF-BB, have been shown to control cellular proliferation and viability,^22^ the effect of xPMC/hPLG on the metabolic activity, doubling time, and viability of periodontal fibroblasts was determined. The results showed that within the first 24 h, cells cultured on xPMC/hPLG exhibited significantly higher metabolic activity and a shorter population doubling time compared with those cultured on hPLG (Figure 4A,B, respectively), suggesting rapid induction of metabolic activity and proliferation. Both conditions also supported further cell growth after 24 h, although no significant differences in metabolic activity or doubling time were observed among these conditions (Figure 4A,B). Moreover, the number of dead cells observed in the xPMC/hPLG group was significantly lower than that observed in the hPLG group at all time points studied (Figure 4C). The results obtained from live/dead cell staining, as shown in Figure 4D, corroborated these findings. While both hPLG and xPMC/hPLG helped increase cell proliferation over 3 days, the proliferative rate after Day 2 in the xPMC/hPLG group was lower than that observed in the hPLG group. This observation was likely consistent with the established inverse relationship between cellular proliferation and differentiation, specifically the premature differentiation-associated cell cycle arrest.^72,73^ Taken together, the results indicated that cells seeded on the xPMC/hPLG hydrogel exhibited superior metabolic activity, a shorter population doubling time, and improved cell viability, compared with those cultured on the hPLG hydrogel.

**Figure 4:**
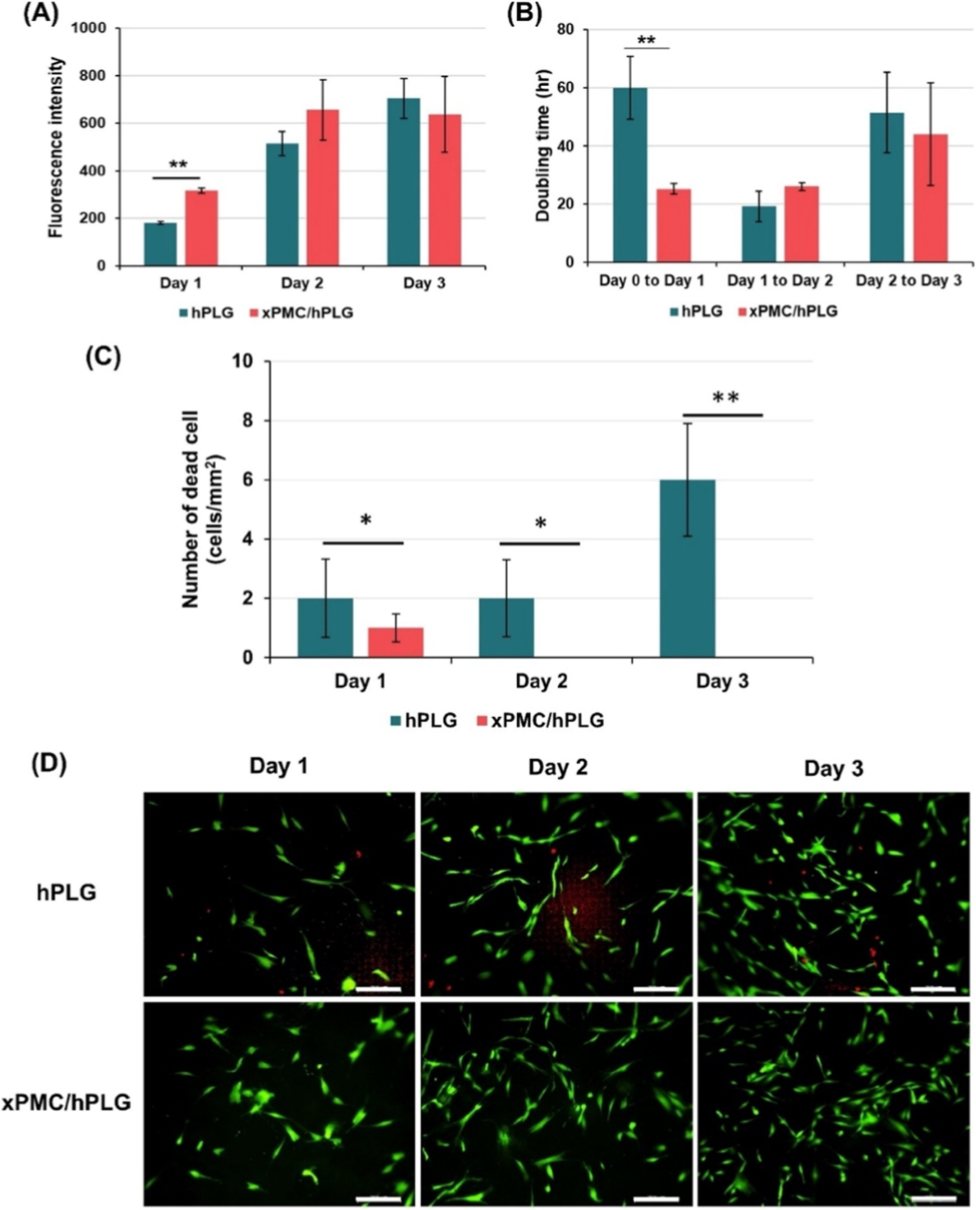
Effect of xPMC/hPLG on the metabolic activity, doubling time, and viability of periodontal fibroblasts. Cells were cultured on hPLG and xPMC/hPLG for 3 days, and (A) the cellular metabolic activity, (B) doubling time, and (C,D) cell viability were determined every 24 h. The results in (A–C) are expressed as mean ± SD (*n* = 3). (D) Shows representative live/dead cell staining where live cells were stained green, and dead cells were stained red. Scale bar = 200 μm.

The concentration-dependent effect of fibrinogen-depleted human platelet lysate (FD-hPL) on the metabolic activity and viability of periodontal fibroblasts was also investigated. As revealed in Figure 5A, the metabolic activity of cells cultured in the 10% FD-hPL-supplemented medium was significantly higher than that of cells treated without FD-hPL (control) and with 25% FD-hPL. Moreover, the greater the FD-hPL concentration was added (50% and 75%), the lower the cellular metabolic activity was observed, which may lead to reduced cell viability. Figure 5B demonstrates the concentration-dependent effect of FD-hPL on the cellular viability assessed using the live/dead cell staining assay. A clear reduction in green-stained live cells and an increase in red-stained dead cells at the two highest concentrations (50% and 75%) confirmed their high toxicity to the periodontal fibroblasts.

**Figure 5:**
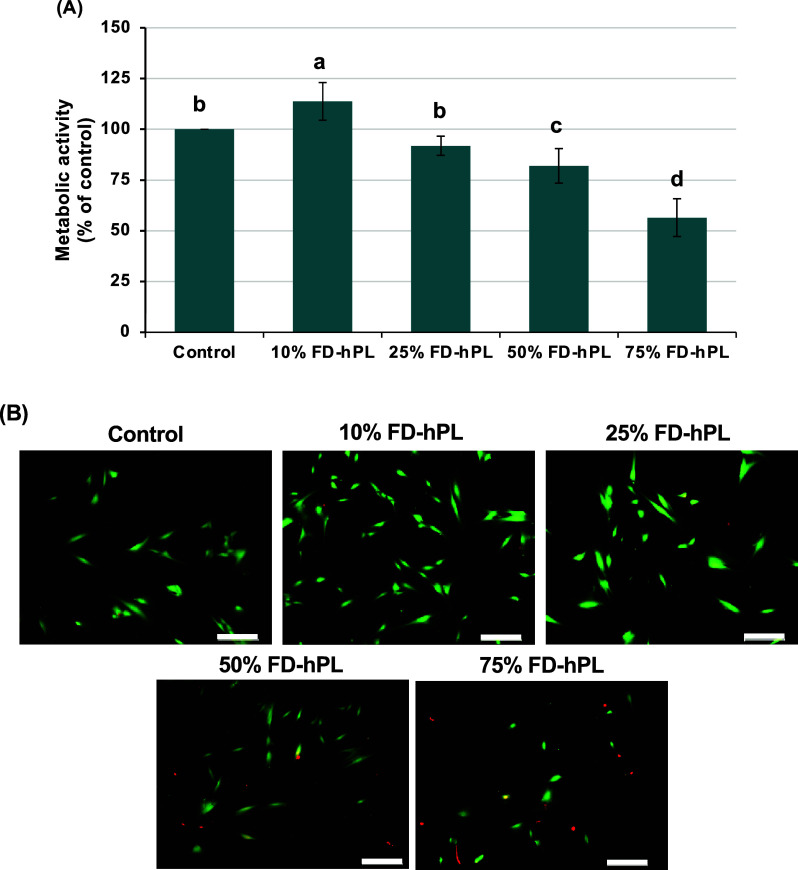
Effect of varied concentrations of FD-hPL on the metabolic activity and viability of periodontal fibroblasts. After the 24 h culture period, the cellular metabolic activity (A) and live/dead cell staining (B) were assessed. In (A), the results are expressed as the mean percentage of viable cells ±SD (*n* = 3), defined as 100% viability in the control group. Different letters represent significant differences (*p* < 0.05). In (B), live cells were stained green, and dead cells were stained red. Scale bar = 200 μm.

While hPL demonstrates considerable promise in several cell culture applications, its utility appears limited by concentration-dependent effects. Tancharoen et al. investigated the impact of hPL supplementation at concentrations ranging from 2.5% to 40% and observed a decrease in the viability of human amniotic fluid mesenchymal stem cells at the highest hPL concentration (40%), indicating an optimal hPL concentration range for hAF-MSC culture.^74^ Likewise, our work demonstrated the cytotoxic concentrations (≥50%) of FD-hPL and suggested the noncytotoxic concentrations (10–25%) of FD-hPL; the total protein range, determined by the BCA analysis, of 10–25% FD-hPL was found to be 486–1215 μg, covering the range of total protein released from hPLG (875 μg) and xPMC/hPLG (795 μg) on Day 1 (Figure 3). The present results suggested that the concentration-dependent effect of FD-hPL on the viability of periodontal fibroblasts exhibited inducing, neutral, suppressive, and toxic effects across the low-to-high concentration range, respectively. Given its vast array of GFs, cytokines, chemokines, vitamins, and other bioactive molecules essential for cell proliferation, migration, and differentiation, hPL, similar to other biologics, exhibited dose-dependent effects.^75–77^ Consequently, an optimal concentration range is typically observed for promoting each specific cellular activity. For example, compared with 3% hPL, 10% hPL supplement supported a stronger induction effect on cell proliferation and migration, but not differentiation, of Wharton’s jelly derived stromal cells.^78^ In contrast, overstimulation by very high hPL concentrations could lead to cytotoxicity.^79^ At nontoxic concentrations above the optimal growth concentration, hPL provided high GFs and nutrients, resulting in an optimal differentiation-promoting effect.^80,81^ Collectively, the effective delivery of hPL, particularly within a biomimetic construct that minimizes processing steps and ensures a slow but optimal release of GFs, is paramount for accelerating clinical translation in chronic wound repair applications.

### 3.5 Migration of Periodontal Fibroblasts

The results of the analysis of cell migration in Figure 6A­(i) showed a sparse density of cells homogeneously surrounding the human platelet-poor plasma gel (hPPG) spot. In contrast, higher cell densities were observed at the peripheries of both hPLG and xPMC/hPLG gel spots, with the greatest density of migrating cells surrounding the xPMC/hPLG gel spot (Figure 6A­(ii,iii)). This confirmed the cell migration-inducing role of hPLG and indicated the added value of xPMC incorporation on cell migration. Among biological mediators present in hPL, PDGF/PDGF receptor signaling, particularly that induced by PDGF-BB, is predominantly effective in inducing cell migration.^22,82^ To verify that PDGF-BB mediated the migration of cells, we experimented using a neutralizing antibody specifically against PDGF-BB.^51,83^ The results revealed that while the treatment with IgG isotype control did not change the migration pattern of cells induced by the xPMC/hPLG gel spot, preincubation with a neutralizing antibody against PDGF-BB resulted in an evenly sparse cell distribution at the spot periphery, comparable to that observed in the hPPG group (Figure 6A­(iv,v)). This indicated that PDGF-BB suppression almost completely inhibited cell migration induced by components present in the lysed platelets, but not the plasma alone. The results of quantitative analysis of cell migration presented in Figure 6B disclosed that complete inhibition of the xPMC/hPLG gel spot–induced cell migration was observed when being treated with a neutralizing antibody against human PDGF-BB. Although IgG isotype control treatment decreased the number of migrating cells present at the xPMC/hPLG spot, this effect was likely attributed to nonspecific binding. Such nonspecific interactions could occasionally lead to the physical obstruction of PDGF-BB from binding to its receptor, consequently diminishing the amount of PDGF-BB that can activate the receptor.^84,85^ Collectively, the results demonstrated that PDGF-BB released from the xPMC/hPLG hydrogel retained its biological activity in stimulating cell migration.

**Figure 6:**
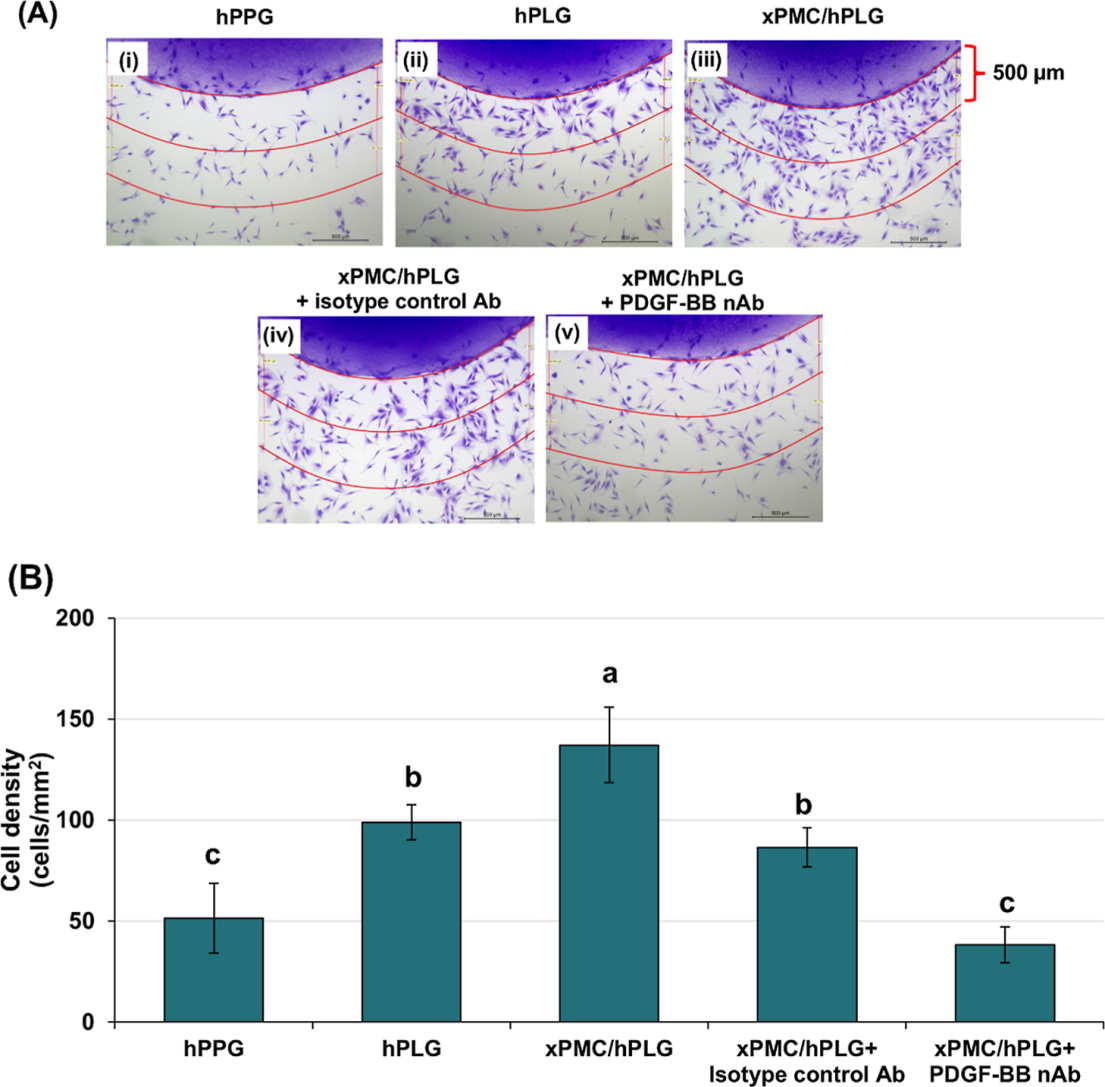
Effect of xPMC/hPLG on the migration of periodontal fibroblasts. (A) Cells were seeded and exposed to chemokines from different gel spots for 6 h. The migratory cells surrounding the edge of gel spots of (i) hPPG, (ii) hPLG, (iii) xPMC/hPLG, (iv) xPMC/hPLG pretreated with IgG isotype control antibody (Ab), and (v) xPMC/hPLG pretreated with neutralizing antibody (nAb) against PDGF-BB were stained with crystal violet (A), and the cell density (cells/mm^2^) was quantified from the number of cells that migrated close to each gel spot within a 500 μm radial distance from the gel edge (B). The results are expressed as mean ± SD (*n* = 8). Different letters indicate significant differences (*p* < 0.05).

Among the GFs present in hPL, PDGF-BB has demonstrated superior efficacy as a migratory cytokine.^22,82^ Although hPLG released a larger quantity of PDGF-BB than xPMC/hPLG (Figure 3D), the latter exhibited more effective cell migration. This was likely because its PDGF-BB slow–release profile achieved an optimal concentration, which was more conducive to the migration of periodontal fibroblasts. This observed migration pattern aligned with prior studies demonstrating the chemotactic effect of hPL on primary human epidermal keratinocytes.^16^ The migration-inducing effect in this study was noted when hPL was used at 10–15%, but not at 20%. More specifically, the PDGF-BB concentration-dependent effect on cell migration has also been reported in metanephric mesenchymal cells, which demonstrated an optimal concentration of 10 ng/mL with a decrease in migration observed at 20 ng/mL PDGF-BB.^85^

The present observations do not exclude the potential contribution of TGF-β1, which may directly or indirectly stimulate cell migration. For instance, Kwon et al. reported that TGF-β1 stimulated the migration of periodontal fibroblasts through the activation of heat shock protein 27.^86^ However, this work demonstrated that the xPMC/hPLG hydrogel controllably released both TGF-β1 and PDGF-BB. This suggested its potential therapeutic value in both preventing the rapid diffusion of these factors from the wound site and increasing migratory resident cells in vivo. Future in vivo studies are undoubtedly needed to determine the full therapeutic benefit of this approach for treating chronic wounds.

The concentration-dependent biphasic effect of PDGF-BB on cell migration and proliferation has been well established.^87–89^ In human saphenous vein smooth muscle cells, chemotactic signal induced by PDGF was dominated by PDGF β-receptors (PDGFRβ) with PDGF-BB being the most effective, and PDGFRβ-mediated chemotactic signal switched from positive at low concentrations of PDGF-BB (1 and 10 ng/mL) to negative at higher concentrations.^89^ Stimulation of cell proliferation was obtained when treating cells with 10–100 ng/mL of PDGF-BB isoforms with similar efficacy to the AB isoform, but not the AA isoform.^89^ The concentration range in which the downturn in chemotaxis began coincided with the range in which cell division was induced. This suggested a potential mechanism by which these cells could utilize a gradient of PDGF-BB to coordinate movement and subsequent proliferation, as proposed in Figure 7. Notably, the xPMC/hPLG-mediated controlled release of endogenous PDGF-BB, present in hPLG, elicited dual functional effects by mediating the low-concentration-dependent migration of distant cells. Subsequently, it stimulated the proliferation of migratory cells at the site of xPMC/hPLG where PDGF-BB was expected to be present at a higher concentration. The present results demonstrated that PDGF-BB appeared to be the sole mediator of the migration of periodontal fibroblasts, despite the presence of many chemotactic mediators in hPL. PDGF-BB was also reported to be most effective in stimulating MSC migration.^90^ While xPMC/hPLG effectively improved the migration induction of hPLG by controlling the release of PDGF-BB, xPMC/hPLG demonstrated a higher cell proliferation-inducing ability for no longer than 24 h. This could be attributed to the still-too-rapid release of other mitogenic mediators present in the hPLG hydrogel. Further modification of the hydrogel to optimize the release of target mediators will help enhance the induction of cell proliferation while preserving cell migration-inducing activity, thereby facilitating new tissue formation.

**Figure 7:**
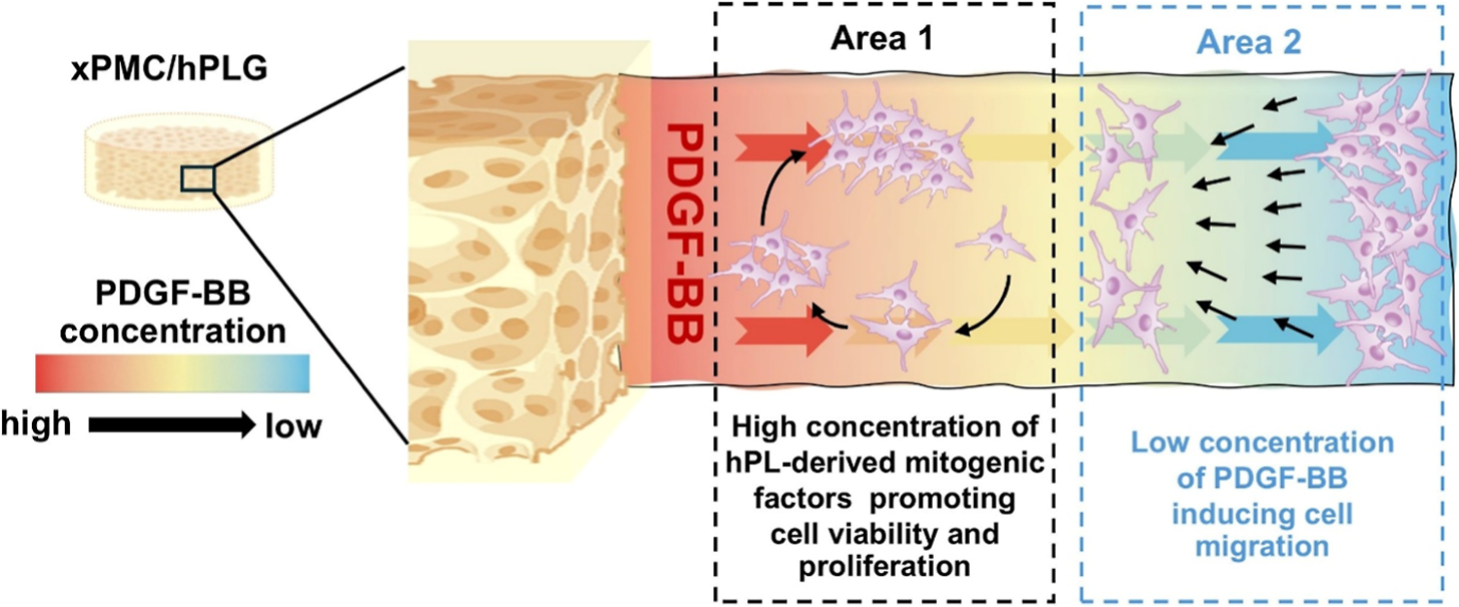
Schematic image of the proposed concentration-dependent biphasic effect of xPMC/hPLG on the chemotaxis and cell doubling of periodontal fibroblasts. Color gradient represents a concentration gradient from high (red) to low (blue) concentrations of hPL-derived mediators released from the xPMC/hPLG hydrogel. The hydrogel gave rise to a concentration gradient of a GF, i.e., PDGF-BB, declining with increasing distance away from the hydrogel. Fibroblasts in Area 2 were exposed to a low concentration of PDGF-BB sufficient to induce their migration toward the hydrogel. As the cells ascended the concentration gradient, the signal for chemotaxis was switched off, but the signal for cell division was switched on, thereby resulting in the presence of proliferating cells at Area 1, adjacent to the hydrogel.

In this study, the xPMC/hPLG composite hydrogel was formulated by embedding xPMC, composed of xCMC and plasma-treated MCM-41 mesoporous silica nanoparticles, in hPL upon its gelation by CaCl_2_. hPL is a promising candidate for regenerative medicine due to its rich and diverse array of GFs and cytokines, which collectively promote wound healing both in vitro and in vivo.^16,91–94^ Compared to other platelet-eluted products, such as PRP and platelet-rich fibrin (PRF), hPL offers distinct advantages, primarily due to its preparation method, which ensures the immediate availability of these potent biomolecules.^95^ As a cell-free preparation, hPL inherently minimizes the risk of cellular immune reactions or inflammation commonly associated with intact cells present in PRP.^18^ This cell-free nature also contributes to reduced variability, a frequent challenge with PRP due to differences in individual donors and preparation protocols.^18^ Furthermore, hPL can be stored long-term without compromising its GF efficacy, facilitating the development of “off-the-shelf” or pooled allogeneic batches.^96^ Meanwhile, CMC is highly biocompatible and readily interacts with proteins, particularly those with a negative charge. It can potentially form networks with fibrin, thereby enhancing the structural integrity of the extracellular matrix within the wound environment. MCM-41 mesoporous silica nanoparticles possess a high surface area, making them ideal for the localized loading of specific antibiotics. This allows for targeted inhibition or eradication of the microorganisms responsible for individual wound infections. Despite its therapeutic potential, the use of hPL is not explicitly FDA-approved. However, its off-label use may be permissible under regulatory exemptions outlined in Title 21 of the United States Code of Federal Regulations, Part 1271 (21 CFR 1271) and the 361-product exemption. It is generally suggested that hPL is exempt when derived from an autologous source, used for homologous tissue purposes, and processed with minimal manipulation.^97^ Similarly, while neither CMC nor MCM-41 is specifically listed as FDA-approved for medical devices or pharmaceutical applications, they are generally recognized as safe (GRAS) in certain contexts, such as food and cosmetics.^31,32^ Consequently, the clinical translation of this hydrogel formulation presents regulatory challenges. Further studies are imperative to finalize the formulation and acquire the necessary safety data to support its application in chronic wound treatment.

## 4 Conclusions

The development of advanced wound dressings capable of stabilizing GFs and modulating the wound microenvironment is critical for treating chronic nonhealing wounds. This study successfully engineered a novel composite hydrogel, xPMC/hPLG, which integrated a highly interconnected porous xPMC component into hPLG. The present results demonstrated a dual functional advantage of xPMC/hPLG. First, the incorporation of xPMC significantly stabilized the hydrogel structure by altering the hPL-derived fibrin network architecture, resulting in a reduced rate of fibrin degradation compared with hPLG alone. Second, the embedded xPMC served as a controlled-release reservoir, thereby optimizing the bioavailability of encapsulated GFs. This controlled-release capability was vital, as the GFs, specifically the potent chemoattractant PDGF-BB, were released at effective concentrations to exert significant chemoattractive effects, simultaneously recruiting distant resident cells and enhancing the proliferation of migratory cells at the wound site. The xPMC/hPLG composite hydrogel showed substantial promise for the treatment of chronic wounds by overcoming the limitations of rapid degradation and insufficient GF efficacy often encountered with single-component biomaterials.

## Supplementary Material



## Notes

Inverted fluorescence microscopy images of human platelet lysate gel (hPLG) autofluorescence (PDF)
