# Supplementary material for: In Vitro Enhanced Performance of Human Platelet Lysate Gel Integrated with Mesoporous Silica Nanoparticle/Carboxymethyl Chitosan Composite Hydrogel: Structural Stability and Biological Activities for Chronic Wound Healing
Source: ACS Omega. 2026 Mar 9;11(11):18241–56. doi: 10.1021/acsomega.5c13494 (PMC13019196; doi:10.1021/acsomega.5c13494)
Supplement: Supplementary file 1 [file ao5c13494_si_001.pdf]

Supporting Information

In Vitro Enhanced Performance of Human Platelet Lysate Gel Integrated with Mesoporous Silica Nanoparticle/Carboxymethyl Chitosan Composite Hydrogel: Structural Stability and Biological Activities for Chronic Wound Healing

Tareerat Lertwimol[a], Suwitchaya Jankam[b], Setthawut Kitpakornsanti[b], Weerachai Singhatanadgit[b,*], and Wanida Janvikul[a,*]

[a] National Metal and Materials Technology Center, National Science and Technology Development Agency, Pathum Thani 12120, Thailand

[b] Faculty of Dentistry and Research Unit in Mineralized Tissue Reconstruction, Thammasat University (Rangsit Campus), Pathum Thani 12121, Thailand

*Authors for correspondence

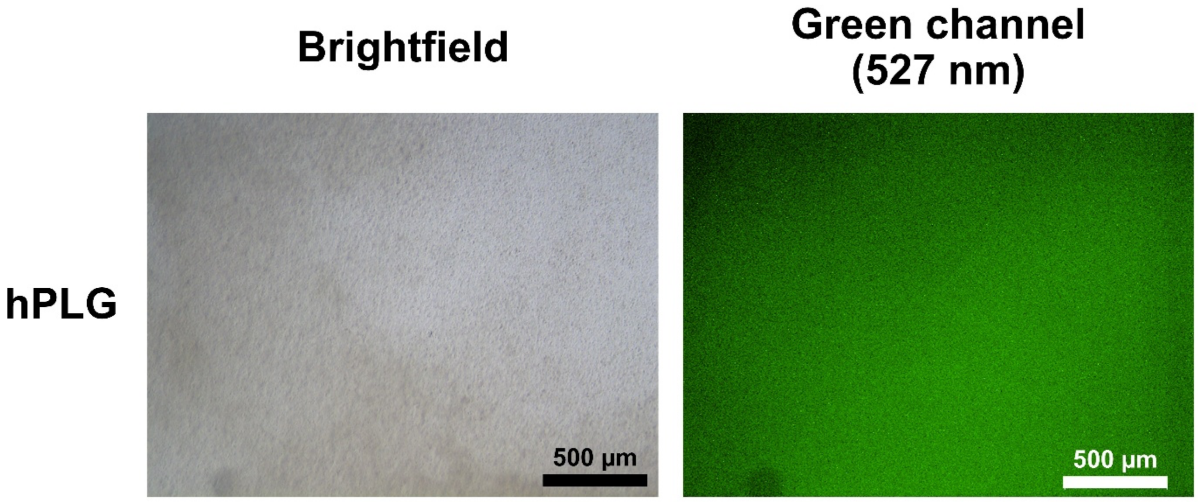

Figure S1. Inverted fluorescence microscopic images (5× objective) of human platelet lysate gel (hPLG) autofluorescence, showing brightfield and green emission channels.
